# Spectrum of gene mutations identified by targeted next‐generation sequencing in Chinese leukemia patients

**DOI:** 10.1002/mgg3.1369

**Published:** 2020-07-07

**Authors:** Hongxia Yao, Congming Wu, Yueqing Chen, Li Guo, Wenting Chen, Yanping Pan, Xiangjun Fu, Guyun Wang, Yipeng Ding

**Affiliations:** ^1^ Department of Hematology Hainan General Hospital (Hainan Affiliated Hospital of Hainan Medical University) Haikou Hainan P.R. China; ^2^ Hainan General Hospital University of South China Haikou Hainan China; ^3^ Department of General Practice Hainan General Hospital (Hainan Affiliated Hospital of Hainan Medical University) Haikou Hainan P.R. China

**Keywords:** gene ontology, INDELs, Leukemia, pathway analysis, SNVs

## Abstract

**Background:**

Despite targeted sequencing have identified several mutations for leukemia, there is still a limit of mutation screening for Chinese leukemia. Here, we used targeted next‐generation sequencing for testing the mutation patterns of Chinese leukemia patients.

**Methods:**

We performed targeted sequencing of 504 tumor‐related genes in 109 leukemia samples to identify single‐nucleotide variants (SNVs) and insertions and deletions (INDELs). Pathogenic variants were assessed based on the American College of Medical Genetics and Genomics (ACMG) guidelines. The functional impact of pathogenic genes was explored through gene ontology (GO), pathway analysis, and protein–protein interaction network in silico.

**Results:**

We identified a total of 4,655 SNVs and 614 INDELs in 419 genes, in which *PDE4DIP*, *NOTCH2*, *FANCA*, *BCR*, and *ROS1* emerged as the highly mutated genes. Of note, we were the first to demonstrate an association of *PDE4DIP* mutation and leukemia. Based on ACMG guidelines, 39 pathogenic and likely pathogenic mutations in 27 genes were found. GO annotation showed that the biological process including gland development, leukocyte differentiation, respiratory system development, myeloid leukocyte differentiation, mesenchymal to epithelial transition, and so on were involved.

**Conclusion:**

Our study provided a map of gene mutations in Chinese patients with leukemia and gave insights into the molecular pathogenesis of leukemia.

## Graphical abstract

We identified a total of 4,655 single‐nucleotide variants and 614 insertions and deletions in 419 genes, in which PDE4DIP, NOTCH2, FANCA, BCR and ROS1 emerged as the highly mutated genes. Based on American College of Medical Genetics and Genomics guidelines, 39 pathogenic and likely pathogenic mutations in 27 genes were found. Our study provided a map of gene mutations in Chinese patients with leukemia and gave insights into the molecular pathogenesis of leukemia.

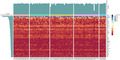

## 1 INTRODUCTION

Leukemia is malignant disorders of the blood and bone marrow (Musharraf, Siddiqui, Shamsi, Choudhary, & Rahman, 2016). In China, there were 75,300 new cases of leukemia and 53,400 death of leukemia in 2015 (Chen et al., 2016). The incidence and mortality of leukemia in males is higher than those in females, and myeloid leukemia has significantly higher levels of incidence and mortality than lymphoid leukemia (Liu, Zhao, Chen, & Chen, 2013). Acute myeloid leukemia (AML), acute lymphoblastic leukemia (ALL), acute promyelocytic leukemia (APL), chronic myeloid leukemia (CML), and chronic lymphocytic leukemia (CLL) are the common types of leukemia (Juliusson & Hough, 2016). Acute leukemia is composed of primary undifferentiated cells; while chronic leukemia, the malignant cells are more differentiated. Exposure to environmental radiation and solvents were reported as predisposing factors for leukemia (Schuz & Erdmann, 2016). However, the direct cause of leukemia has not been found. Recently, mutation profiling of genes provides prognostic prediction and treatment guidance for patients with leukemia (Itzykson et al., 2013; Shin et al., 2016). Reports on mutational patterns of Chinese leukemia patients are limited.

The application of next‐generation sequencing (NGS) technique can better achieve testing for a larger group of mutational markers. NGS is a massively parallel high‐throughput DNA sequencing approach (Metzker, 2010). The major advantages of this approach are that simultaneously screen a large number of genes and samples using very low amount of nucleic acids, and have high sensitivity for mutation detection (Meldrum, Doyle, & Tothill, 2011). Types of NGS include whole‐genome sequencing, whole‐exome sequencing, whole‐transcriptome sequencing, and targeted regions sequencing (Ross & Cronin, 2011). Targeted regions sequencing for multiple specific genomic regions have been widely employed in many fields to identify genetic variants related to disease pathogenesis and prognosis (Mansouri et al., 2014).

Despite targeted sequencing have identified several mutations for leukemia, there is still a limit of mutation screening for Chinese leukemia. Here, we performed targeted regions sequencing containing these 504 genes in 109 patients with leukemia among Chinese Han population to explore the genetic basis of Chinese leukemia patients.

## 2 MATERIALS AND METHODS

### 2.1 Study population

A total of 109 patients diagnosed with leukemia at Hainan General Hospital were enrolled. All of the patients were genetically unrelated ethnic Han Chinese. Patients with leukemia were diagnosed according to 2016 WHO classification criteria (Sabattini, Bacci, Sagramoso, & Pileri, 2010). The samples were confirmed by bone marrow microscopy, flow immunophenotyping, chromosome screening, and fusion gene detection. Our study was approved by the ethical review board of Hainan General Hospital, and complied with the Declaration of Helsinki. Informed written consent was obtained from all patients enrolled in this study.

### 2.2 DNA extraction and sequencing

Peripheral blood (5 ml) was collected from each subject into EDTA‐coated vacutainer tubes. Genomic DNA was isolated using a commercially available DNA extraction kit (GoldMag Co. Ltd.), and then quantified using a NanoDrop 2000 Spectrophotometer (NanoDrop Technologies).

The sample prepared using a Truseq DNA Sample preparation Kit (Illumina) following the standard protocol. Agilent SureDesign website (https://earray.chem.agilent.com/‐suredesign/home.htm) was used to design capture oligos for 504 cancer‐related genes. Paired‐end libraries were prepared following the Illumina protocol. Hybridization reactions were performed on AB 2720 Thermal Cycler (Life Technologies Corporation). The hybridization mixture was captured using magnetic beads (Invitrogen) and Agilent Custom Sureselect Enrichment Kit according to the manufacturer's instructions. Sequencing (2 × 150 bp reads) was carried out on Illumina HiSeq2500 platform (Illumina).

Sequencing reads were aligned to the human reference genome (UCSC Genome Browser hg19, http://genome.ucsc.edu/) using the Burrows‐Wheeler Aligner (Li & Durbin, 2010). Picard software (https://github.com/broadinstitute/picard) was employed to remove duplicate PCR reads and evaluate the quality of variants by attaining effective reads, effective base, and average coverage depth. Sequencing quality controls were as follows: (a) Q20 and Q30 more than 90% and 80%, respectively; (b) coverage of target regions more than 99%; and mapping rate no less than 95%. single‐nucleotide variant (SNV) calling was performed using GATK and Varscan programs (Koboldt et al., 2012; McKenna et al., 2010). Variants were annotated using ANNOVAR (http://annovar.openbioinformatics.org/en/latest/).

### 2.3 In silico analysis

Pathogenic or likely pathogenic SNVs was assessed by the 1000 Genomes Project, Sorting Tolerant From Intolerant (SIFT), PolyPhen, MutationTaster, and Combined Annotation Dependent Depletion (CADD) based on the American College of Medical Genetics and Genomics (ACMG) guidelines (Richards et al., 2015). Pathway enrichment analysis of gene ontology (GO) and the Kyoto Encyclopedia of Genes and Genomes (KEGG) for the candidate pathogenic genes was carried out using R clusterprofiler software package (http://bioconductor.org/packages/release/bioc/html/clusterProfiler.html). GO terminology was annotated on the following three aspects: cellular component (GO‐CC), molecular function (GO‐MF), and biological process (GO‐BP). Pathway enrichment based on KEGG pathway database (https://www.kegg.jp/kegg/) was applied for pathway annotation. GO terms and KEGG pathway with *p* < .05 were considered to be significant. Protein–protein interaction (PPI) network was predicted by STRING online software (https://string‐db.org/). GEPIA (http://gepia.cancer‐pku.cn/) database was used to evaluate the expression and prognostic of candidate genes in leukemia.

## 3 RESULTS

### 3.1 Clinical characteristics of patients with leukemia

A total of 109 patients with leukemia were identified. The median age of the studied cohort was 39.83 years (range: 11–77 years), included 58 (53.2%) males and 51 (46.8%) females (Table 1). Among these, 30 patients diagnosed as AML, 19 patients as ALL, 24 patients as APL, 28 patients as CML, and 8 patients as CLL.

**Table 1:** Characteristics of patients with leukemia

Variable	Total (*n* = 109)	AML (*n* = 30)	ALL (*n* = 19)	APL (*n* = 24)	CML (*n* = 28)	CLL (*n* = 8)
Age, years	39.8 (11–77)	29.5 (12–74)	36 (13–73)	41 (16–62)	42 (11–75)	60.5 (51–77)
Gender						
Male, *n*	58	13	10	11	18	6
Female, *n*	51	17	9	13	10	2
SNVs and INDELs						
SNVs, *n*	4,655	2,435	1,870	2,570	2,701	2,661
Indels, *n*	614	359	309	388	408	393
Blast (%, IQR)	16.00 (2.50–47.50)	36.50 (26.25–59.00)	47.50 (14.00–80.00)	2.25 (1.50–2.88)	1.25 (1.00–4.50)	3.00 (2.13–6.75)
Lymphocytes population (%, IQR)	16.00 (6.65–41.42)	13.60 (8.18–18.43)	23.90 (14.90–44.30)	31.86 (14.28–43.65)	6.90 (1.95–23.4)	79.25 (64.00–86.18)
BCR‐ABL1 transcript positive (*n*)					24	

Abbreviations: ALL, acute lymphoblastic leukemia; AML, acute myeloid leukemia; APL, acute promyelocytic leukemia; CLL, chronic lymphocytic leukemia; CML, chronic myeloid leukemia; INDELs, insertions and deletions; IQR, interquartile range; SNV, single‐nucleotide variants.

### 3.2 Spectrum of gene variants

**Figure 1:**
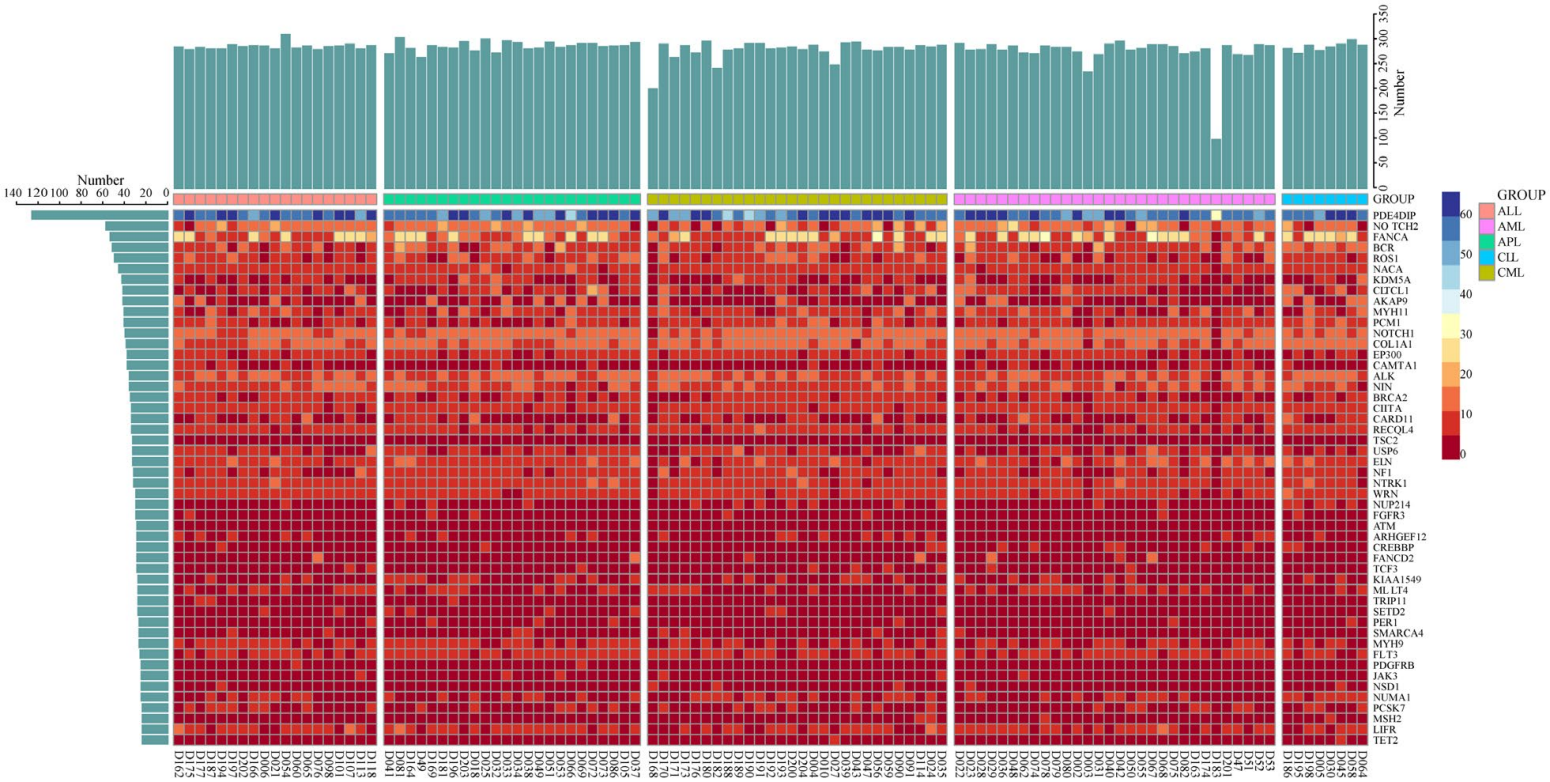
Landscape of mutations in 109 leukemia patients (top 50 mutated genes). The number of SNVs and INDELs for each gene is shown (left). The number of SNVs and INDELs for each patient is shown (top). Each vertical column represented an individual. Shaded bars indicate the number of mutations in genes. INDELs, insertions and deletions; SNVs, single‐nucleotide variants

The targeted capture deep sequencing of 504 tumor‐related genes in 109 leukemia samples revealed 4,655 SNVs and 614 insertions and deletions (INDELs) in 419 genes (Table S1). There were 208 genes with greater than 10 mutations, 78 genes with greater than 20 mutations. Top 50 mutated genes across the leukemia patients were described in Figure 1. Among them, the most commonly mutated gene was *PDE4DIP* (128), followed by *NOTCH2* (59), *FANCA* (55), *BCR* (53), *ROS1* (51), *NACA* (47), *KDM5A* (44), *CLTCL1* (43), *AKAP9* (43), *MYH11* (42), *PCM1* (42), *NOTCH1* (41), *COL1A1* (40), and so on. According to GEPIA database, *PDE4DIP* was down‐regulated in AML compared with normal samples (*p* < .01), and high expression of *PDE4DIP* was associated with poor AML prognosis (HR = 2.3, logrank *p* = .0027, Figure S1). STRING database was used to predict the potential interacting protein with PDE4DIP, as Figure S2 and Table S2. The results showed that PDE4DIP protein might interact with PDE4D, PRKAR2A, and AKAP9, which can bind to cAMP or protein kinase A (PKA), suggesting that PDE4DIP may participate in cAMP/PKA signaling pathway.

**Figure 2:**
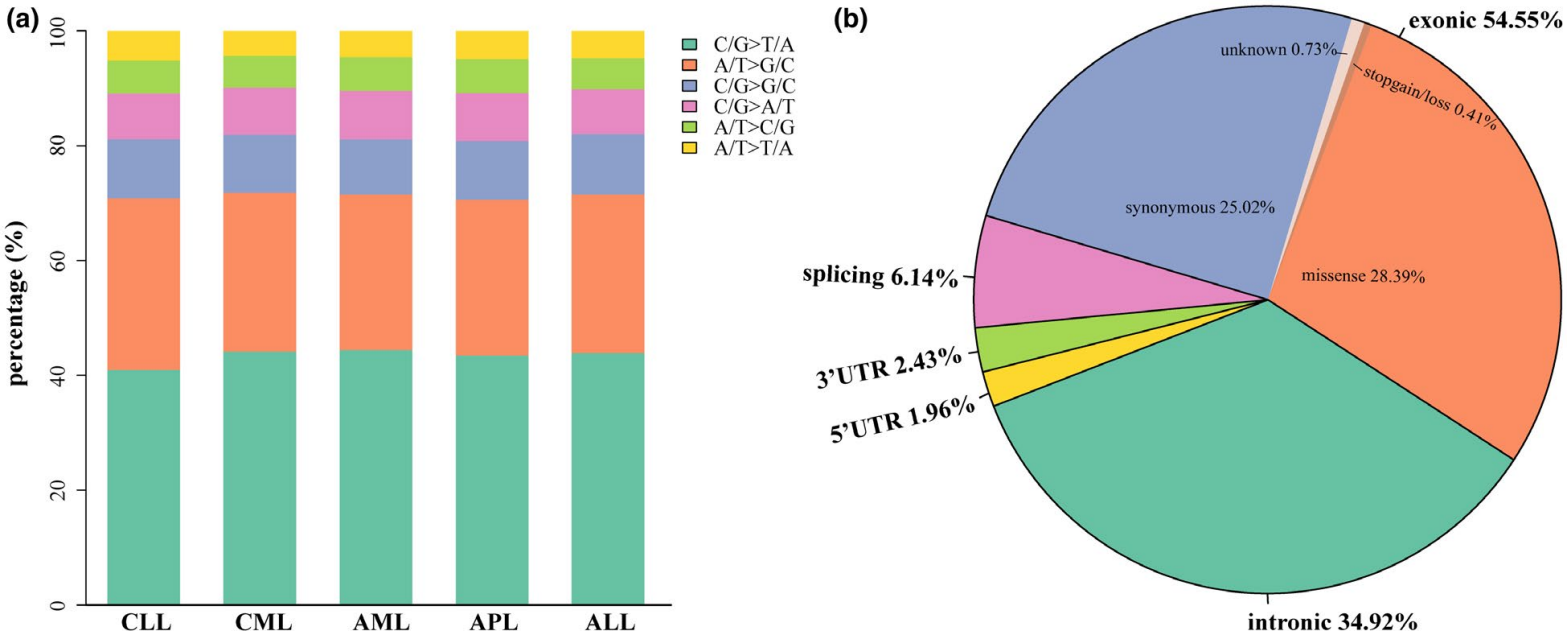
Patterns of somatic SNVs by targeted next‐generation sequencing. (a) The percentages of distinct transitions and transversions of SNVs. (b) Proportions of SNVs types according to their regions in the gene. SNVs, single‐nucleotide variants

We analyzed single‐nucleotide changes of these detected SNVs and found that transitions as C/G>T/A and A/T>G/C were more prevalent than transversions including C/G>G/C, C/G>A/T, A/T>C/G, and A>T/T>A in all leukemia patients, as shown in Figure 2a. In addition, we also analyzed the regions of these SNVs. Among these variants (Figure 2b), exonic variants (54.55%) were the most frequent, followed in order by intronic variants (34.92%), splicing variants (6.14%), and UTR variants (4.39%). Additionally, we detected the genetic effects of these variants in exonic region, with missense variants, synonymous variants, stopgain/loss variants, and unknown variants.

There were 2,435 SNVs and 359 INDELs in AML, 1,870 SNVs and 309 INDELs in ALL, 2,570 SNVs and 388 INDELs in APL, 2,701 SNVs and 408 INDELs in CML, and 2,661 SNVs and 393 INDELs in CLL. We then compared the frequency of mutations and INDELs in five subgroups of leukemia. Mutations or INDELs in 50 genes (*PDE4DIP, NOTCH2, FANCA, BCR, CLTCL1, MYH11, NOTCH1, COL1A1, CAMTA1, ALK, NIN, CIITA, CARD11, RECQL4, USP6, NF1, NUP214, KIAA1549, MYH9, FLT3, NSD1, PCSK7, XPC, PMS2, SETBP1, SRGAP3, ASXL1, PDGFRA, ATIC, RALGDS, PRDM16, TSHR, FGFR2, EGFR, CDH11, DNM2, SYK, MUTYH, BCOR, FNBP1, RAC1, IL7R, CNOT3, RNF43, TP53, KCNJ5, MEN1, SH2B3, SRSF2, CREB3L1*) were shared across all patients with leukemia. The different pathological classification of leukemia might have distinct patterns of variants and INDELs. Table S3 showed the top 50 significantly different genes. In ALL, the mutation frequencies of *XPA*, *CDK4*, and *BCL11B* were higher than other subgroups, but the frequencies of *SMARCE1* and *FEV* were lower. The high prevalence of *GNAQ*, *ELF4*, *HOXD13*, and *COX6C* mutations in patients with APL were noteworthy, *SMARCE1* variant predominated in patients with CML, variant of *PER1* and *ETV6* had higher frequencies in patients with AML. The variants of *PIK3CA*, *CHCHD7*, *FEV*, and *PHF6* variants were significantly enriched in CLL. These data suggested a strong functional role of these genes in the different types of leukemia.

### 3.3 The filtrate pathogenic and likely pathogenic genes

**Figure 3:**
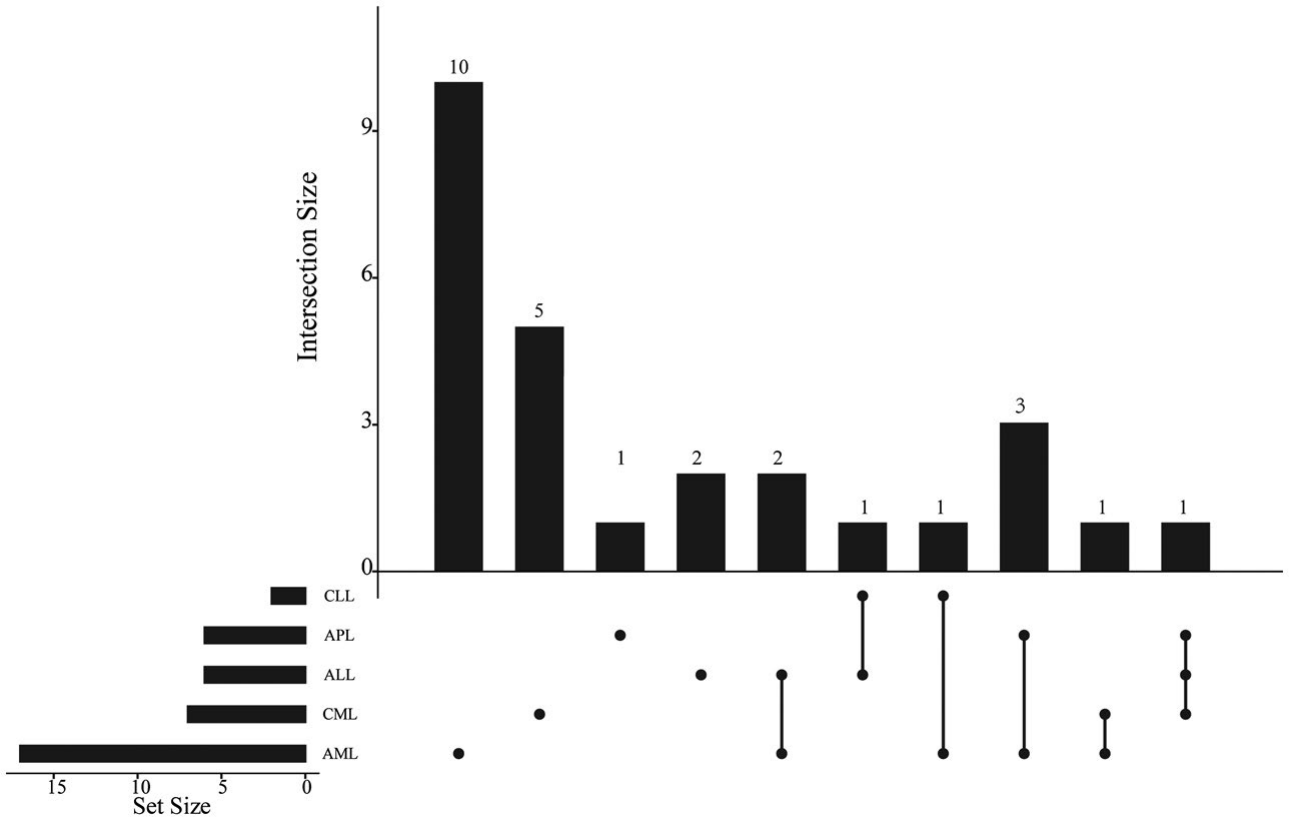
Overview of the distribution of the 27 pathogenic and likely pathogenic gene according to ACMG guidelines in five subgroup of leukemia. ACMG, American College of Medical Genetics and Genomics

Based on ACMG guideline, we identified 39 pathogenic and likely pathogenic mutations in 27 genes including SNVs or INDELs in exonic and splicing regions among 32 leukemia patients (Figure 3 and Table 2). In AML, 21 pathogenic or likely pathogenic germline mutations in 17 genes were identified, of which 10 mutant genes only in AML patients. ALL patients had six pathogenic or likely pathogenic germline mutations, especially *PBRM1* (c.2819_2829del, p.L940fs) and *SUZ12* (c.1716_1717insG, p.L572fs). There were six pathogenic or likely pathogenic germline mutations among APL patients, of which *PAX8* (c.G201T, p.E67D) only in APL patients. Two pathogenic mutations in *ALDH2* (rs540073928, p.A175D) and *FBXW7* (rs866987936, p.R361Q) were found in CLL patients. Among CML patients, seven pathogenic or likely pathogenic germline mutations were also detected, of which five mutant genes just in CML patients. These results hinted us that these mutations might play an important role in the pathogenesis of the different leukemia subgroup. The pathologic mutations for leukemia patients were shown in Table S4.

**Table 2:** Summary of ACMG Likely Pathogenic mutations

Gene	Leukemia	Priority	Chr: Position	SNV/Indel	REF	ALT	Region	Transcript ID	Exonic Function	Nucleotide change	AA change
ALDH2	AML, CLL	H	12:112228350	rs540073928	C	A	exonic	NM_001204889	missense SNV	c.C524A	p.A175D
ASXL1	AML	H	20:31021634	.	C	CA	exonic	NM_015338	frameshift insertion	c.1633_1634insA	p.R545fs
ATIC	AML	H	2:216182882	rs575560797	A	T	exonic	NM_004044	missense SNV	c.A149T	p.D50V
CANT1	AML	H	17:76993297	.	CA	C	exonic	NM_001159772	frameshift deletion	c.407delT	p.L136fs
CBL	AML	H	11:119155730	.	C	CCGCGCTTTCTT	exonic	NM_005188	frameshift insertion	c.1483_1484insCGCGCTTTCTT	p.P495fs
CBL	CML	H	11:119148892	rs387906666	A	G	exonic	NM_005188	missense SNV	c.A1112G	p.Y371C
CEBPA	AML	H	19:33793130	.	AT	A	exonic	NM_00128743	frameshift deletion	c.295delA	p.I99fs
CEBPA	AML	H	19:33793153	.	GCAGATGCCGCC	G	exonic	NM_001287435	frameshift deletion	c.262_272del	p.G88fs
CEBPA	APL	H	19:33793092	.	A	ACT	exonic	NM_001287435	frameshift insertion	c.333_334insAG	p.F112fs
FANCG	CML	H	9:35077398	rs376732298	T	TG	splicing	NM_004629	.		
FBXW7	ALL	H	4:153249385	rs867384286	G	A	exonic	NM_018315	missense SNV	c.C1039T	p.R347C
FBXW7	CLL	H	4:153247366	rs866987936	C	T	exonic	NM_018315	missense SNV	c.G1082A	p.R361Q
FLT3	AML, APL	H	13:28592642	rs121913488	C	A	exonic	NM_004119	missense SNV	c.G2503T	p.D835Y
FLT3	AML	H	13:28592640	rs121913487	A	C	exonic	NM_004119	missense SNV	c.T2505G	p.D835E
FOXP1	CML	H	3:71037162	.	TG	T	exonic	NM_001244810	frameshift deletion	c.828delC	p.P276fs
IDH1	AML	H	2:209113113	rs121913499	G	C	exonic	NM_001282387	missense SNV	c.C394G	p.R132G
KIAA1549	AML	H	7:138602332	.	TGA	T	exonic	NM_020910	frameshift deletion	c.2038_2039del	p.S680fs
KRAS	AML	H	12:25398281	rs112445441	C	T	exonic	NM_004985	missense SNV	c.G38A	p.G13D
MYH9	AML	H	22:36715582	rs372016779	T	C	splicing	NM_002473	.		
NFIB	CML	H	9:14398522	.	C	A	splicing	NM_001190738	.		
NRAS	AML	H	1:115258744	rs121434596	C	A	exonic	NM_002524	missense SNV	c.G38T	p.G13V
NRAS	AML	H	1:115256529	rs11554290	T	G	exonic	NM_002524	missense SNV	c.A182C	p.Q61P
NRAS	ALL	H	1:115258747	rs121913237	C	T	exonic	NM_002524	missense SNV	c.G35A	p.G12D
PAX5	AML	H	9:36966685	.	AGCGAGTG	A	exonic	NM_001280552	frameshift deletion	c.310_316del	p.H104fs
PAX8	APL	H	2:114002192	.	C	A	exonic	NM_013953	missense SNV	c.G201T	p.E67D
PBRM1	ALL	H	3:52623221	.	GTAAGCCTGAGA	G	exonic	NM_018313	frameshift deletion	c.2819_2829del	p.L940fs
RUNX1	CML	H	21:36171607	.	G	A	exonic	NM_001001890	stopgain	c.C877T	p.R293X
SETBP1	CML	H	18:42531913	rs267607040	G	A	exonic	NM_015559	missense SNV	c.G2608A	p.G870S
SETD2	AML	H	3:47155452	.	C	CCGGTCCAA	exonic	NM_014159	frameshift insertion	c.4628_4629insTTGGACCG	p.R1543fs
SMO	AML	H	7:128846423	.	T	C	exonic	NM_005631	missense SNV	c.T1259C	p.I420T
SMO	ALL	H	7:128848655	.	G	C	exonic	NM_005631	missense SNV	c.G1320C	p.K440N
SUZ12	ALL	H	17:30322703	.	A	AG	exonic	NM_015355	frameshift insertion	c.1716_1717insG	p.L572fs
TFRC	ALL, APL	H	3:195791279	rs184956956	C	T	exonic	NM_003234	missense SNV	c.G1219A	p.A407T
TFRC	CML	H	3:195785460	rs772017482	T	C	exonic	NM_003234	missense SNV	c.A1580G	p.N527S
TP53	AML	H	17:7578406	rs28934578	C	T	exonic	NM_001126115	missense SNV	c.G128A	p.R43H
WT1	APL	H	11:32413557	.	G	T	exonic	NM_024426	missense SNV	c.C1342A	p.H448N
WT1	APL	H	11:32417920	.	G	GAGTCGGGGCTACTCCAGGC	exonic	NM_024426	frameshift insertion	c.1080_1081insGCCTGGAGTAGCCCCGACT	p.L361fs
WT1	AML	H	11:32417909	.	C	CGACA	exonic	NM_024426	frameshift insertion	c.1091_1092insTGTC	p.S364fs
WT1	AML	H	11:32417942	.	A	AG	exonic	NM_024426	frameshift insertion	c.1058_1059insC	p.R353fs

Abbreviations: ACMG, American College of Medical Genetics and Genomics; ALL, acute lymphoblastic leukemia; ALT, alter; AML, acute myeloid leukemia; APL, acute promyelocytic leukemia; CLL, chronic lymphocytic leukemia; CML, chronic myeloid leukemia; INDELs, insertions and deletions; REF, reference; SNV, single nucleotide variants.

### 3.4 GO annotation for pathogenic and likely pathogenic genes

**Figure 4:**
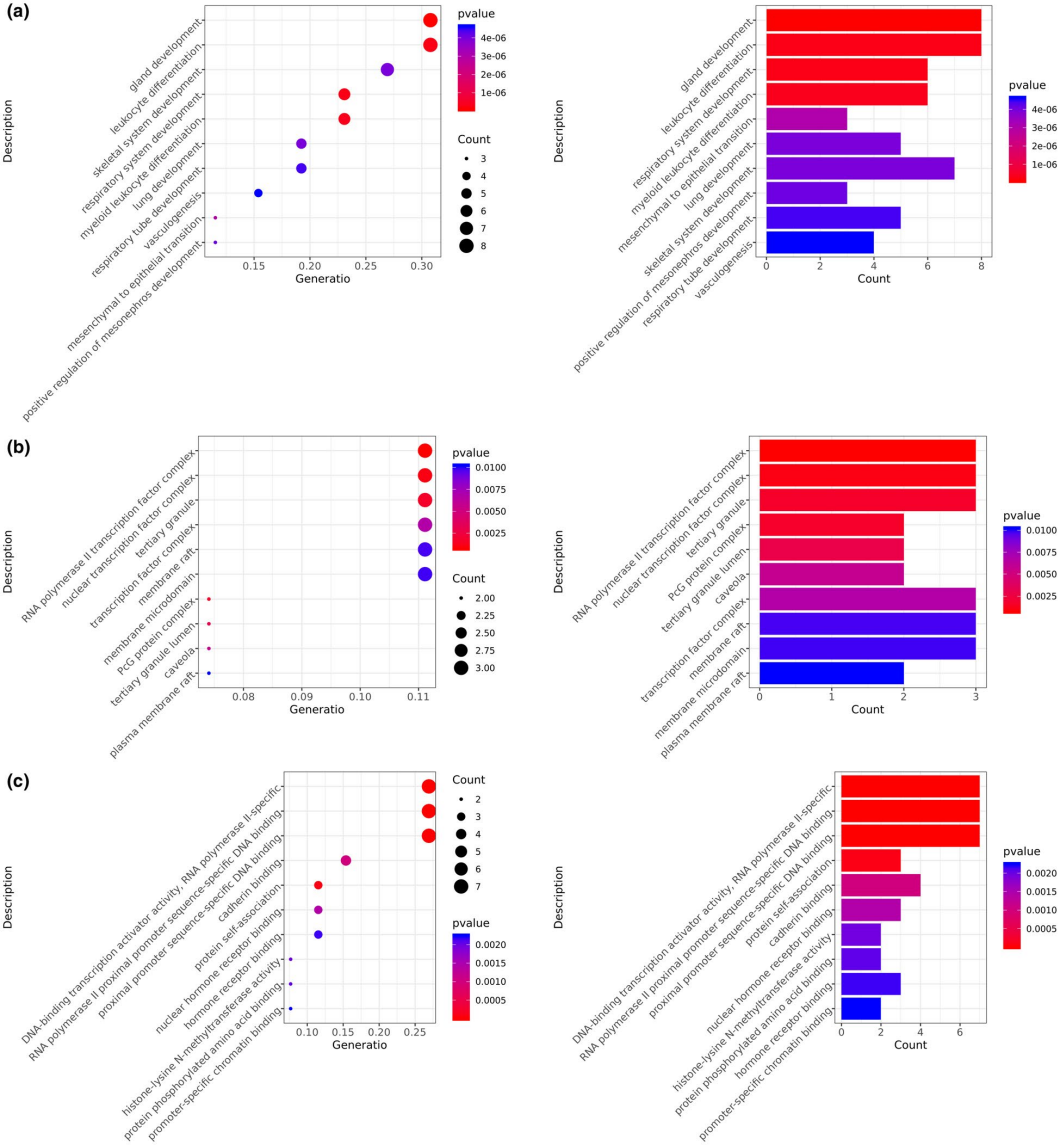
Top 10 enrichment scores in GO enrichment analysis for 27 pathogenic and likely pathogenic genes. (a) Biological process of pathogenic and likely pathogenic genes; (b) Cellular component of pathogenic and likely pathogenic genes; (c) Molecular function of pathogenic and likely pathogenic genes. GO, gene ontology

Gene ontology annotation and pathway analyses were conducted for 27 pathogenic and likely pathogenic genes. The possible BP of these overlapping genes were related to the gland development, leukocyte differentiation, respiratory system development, myeloid leukocyte differentiation, mesenchymal to epithelial transition, lung development, skeletal system development, positive regulation of mesonephros development, respiratory tube development, vasculogenesis, and so on (Figure 4a). The results of GO‐CC annotation suggested that these genes were involved in CC including RNA polymerase II transcription factor complex, nuclear transcription factor complex, tertiary granule, PcG protein complex, tertiary granule lumen, caveola, transcription factor complex, membrane raft, membrane microdomain, and plasma membrane raft (Figure 4b). Moreover, the MF of these pathogenic and likely pathogenic genes were mainly correlated with the DNA‐binding transcription activator activity, RNA polymerase II‐specific, RNA polymerase II proximal promoter sequence‐specific DNA binding, proximal promoter sequence‐specific DNA binding, protein self‐association, cadherin binding, nuclear hormone receptor binding, histone‐lysine *N*‐methyltransferase activity, protein phosphorylated amino acid binding, hormone receptor binding, promoter‐specific chromatin binding (Figure 4c).

### 3.5 Pathway analysis and prediction of PPI

Further enrichment analysis based on the KEGG database showed that these pathogenic and likely pathogenic genes were highly enriched in leukemia and cancer‐related pathways, as shown in Table 3. The pathways included (a) AML and CML; (b) cancer‐related pathways, such as transcriptional misregulation, central carbon metabolism, and proteoglycans; (c) various cancers including thyroid cancer, bladder cancer, and endometrial cancer.

**Table 3:** Annotation of pathways for pathogenic and likely pathogenic genes

ID	Description	Gene Ratio	Bg Ratio	P value	Q value	Gene ID	Count
hsa05221	Acute myeloid leukemia	5/21	57/7010	5.48 × 10^−7^	2.83 × 10^−5^	CEBPA/FLT3/KRAS/NRAS/RUNX1	5
hsa05202	Transcriptional misregulation in cancer	7/21	180/7010	5.62 × 10^−7^	2.83 × 10^−5^	CEBPA/FLT3/PAX5/PAX8/RUNX1/TP53/WT1	7
hsa05200	Pathways in cancer	9/21	397/7010	8.76 × 10^−7^	2.83 × 10^−5^	CBL/CEBPA/FLT3/KRAS/NRAS/PAX8/RUNX1/SMO/TP53	9
hsa05230	Central carbon metabolism in cancer	5/21	67/7010	1.24 × 10^−6^	2.83 × 10^−5^	FLT3/IDH1/KRAS/NRAS/TP53	5
hsa05216	Thyroid cancer	4/21	29/7010	1.35 × 10^−6^	2.83 × 10^−5^	KRAS/NRAS/PAX8/TP53	4
hsa05220	Chronic myeloid leukemia	5/21	73/7010	1.91 × 10^−6^	3.33 × 10^−5^	CBL/KRAS/NRAS/RUNX1/TP53	5
hsa05219	Bladder cancer	3/21	41/7010	0.00023	0.003444	KRAS/NRAS/TP53	3
hsa05205	Proteoglycans in cancer	5/21	203/7010	0.00027	0.003546	CBL/KRAS/NRAS/SMO/TP53	5
hsa05213	Endometrial cancer	3/21	52/7010	0.000466	0.005437	KRAS/NRAS/TP53	3
hsa05223	Non‐small cell lung cancer	3/21	56/7010	0.00058	0.00609	KRAS/NRAS/TP53	3
hsa05214	Glioma	3/21	65/7010	0.000898	0.008575	KRAS/NRAS/TP53	3
hsa05218	Melanoma	3/21	71/7010	0.001162	0.010167	KRAS/NRAS/TP53	3
hsa04012	ErbB signaling pathway	3/21	87/7010	0.002089	0.016725	CBL/KRAS/NRAS	3
hsa05215	Prostate cancer	3/21	89/7010	0.00223	0.016725	KRAS/NRAS/TP53	3
hsa04211	Longevity regulating pathway—mammal	3/21	94/7010	0.002607	0.018248	KRAS/NRAS/TP53	3
hsa04660	T cell receptor signaling pathway	3/21	104/7010	0.003474	0.022799	CBL/KRAS/NRAS	3
hsa04919	Thyroid hormone signaling pathway	3/21	118/7010	0.004957	0.028711	KRAS/NRAS/TP53	3
hsa04722	Neurotrophin signaling pathway	3/21	120/7010	0.005195	0.028711	KRAS/NRAS/TP53	3
hsa04071	Sphingolipid signaling pathway	3/21	120/7010	0.005195	0.028711	KRAS/NRAS/TP53	3
hsa05160	Hepatitis C	3/21	133/7010	0.006916	0.036307	KRAS/NRAS/TP53	3
hsa04530	Tight junction	3/21	139/7010	0.007812	0.036373	KRAS/MYH9/NRAS	3
hsa04910	Insulin signaling pathway	3/21	139/7010	0.007812	0.036373	CBL/KRAS/NRAS	3
hsa04210	Apoptosis	3/21	140/7010	0.007967	0.036373	KRAS/NRAS/TP53	3
hsa05161	Hepatitis B	3/21	146/7010	0.008941	0.039115	KRAS/NRAS/TP53	3

**Figure 5:**
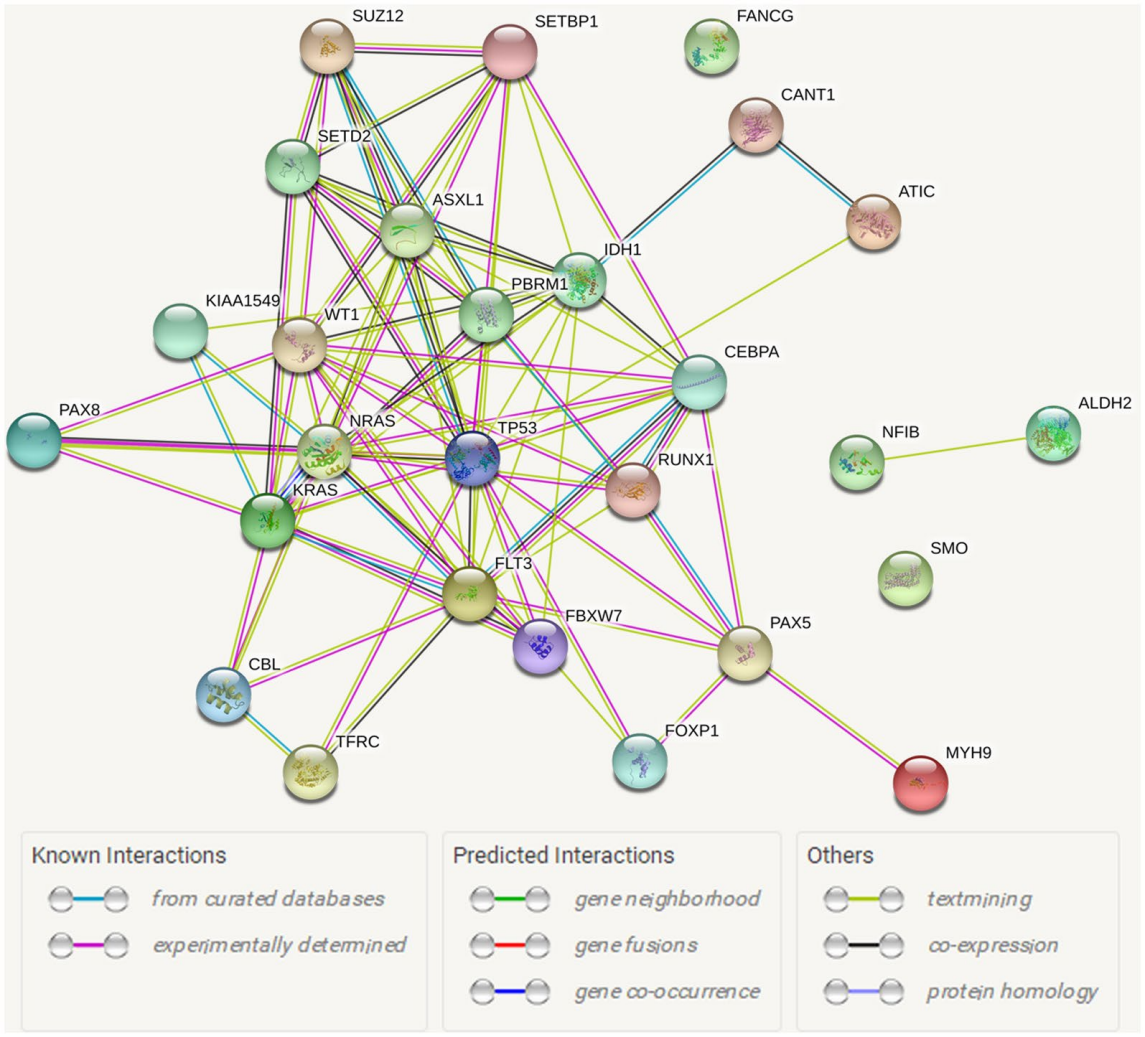
PPI network for 27 pathogenic and likely pathogenic genes. PPI, protein–protein interaction

STRING database was used to construct the PPI network for 27 pathogenic and likely pathogenic genes. String map displayed these genes containing 27 nodes and 88 edges, in which nodes representing proteins and edges depicting associated interactions (Figure 5). PPI analysis showed that CEBPA, FLT3, PAX5, PAX8, RUNX1, TP53, and WT1 genes located in network hub appeared in Transcriptional misregulation in cancer.

## 4 DISCUSSION

In this study, we performed a targeted capture deep sequencing of 504 tumor‐related genes in 109 leukemia samples. We identified a total of 4,655 SNVs and 614 INDELs in 419 genes, in which *PDE4DIP*, *NOTCH2*, *FANCA*, *BCR*, *ROS1*, *NACA*, *KDM5A*, *CLTCL1*, *AKAP9*, *MYH11*, *PCM1*, *NOTCH1*, *COL1A1* had more than 40 mutations. We compared the frequency of mutations and INDELs in five subgroup of leukemia (ALL, APL, AML, CLL, and CML). Moreover, ACMG pathogenic analysis identified 27 pathogenic and likely pathogenic genes. GO enrichment analysis, pathway analysis, and PPI network were performed on the pathogenic and likely pathogenic genes. Our results might provide some molecular data on mutations in leukemia to map the genetic variations of Chinese patients with leukemia.

Mutational analysis was used to map the genetic variants in leukemia, and found that the highest mutations occurred in *PDE4DIP*, followed by *NOTCH2*, *FANCA*, *BCR*, and *ROS1* in almost all leukemia patients. Phosphodiesterase 4D interacting protein (PDE4DIP) anchored PDE4D at the centrosome‐Golgi cell region, which was involved in signal transduction and hydrolyze cGMP and cAMP to energize several reactions in the cell, including related to immune cell activation, hormone secretion, smooth vascular muscle action, and platelet aggregation (Shapshak, 2012). The protein is found to interact with a phosphodiesterase superfamily protein member (Vinayagam et al., 2011). The *PDE4DIP* mutations have also been previously identified in various cancers including lung cancer, medullary thyroid cancer, and ovarian cancer (Chang et al., 2018; Er et al., 2016; Y. Li et al., 2018), but not been reported in leukemia previously. Our study first reported that *PDE4DIP* mutations (128) in leukemia patients, suggesting *PDE4DIP* would be involved in pathogenesis of leukemia. Moreover, we found that *PDE4DIP* was downregulated in AML base on TCGA database, and the high expression was associated with poor AML prognosis. These suggested that PDE4DIP might be a tumor suppressor gene in leukemia. Results of STRING database showed that PDE4DIP protein might interact with PDE4D, PRKAR2A, and AKAP9, which can bind to cAMP or PKA, suggesting that PDE4DIP may participate in cAMP/PKA signaling pathway. In addition, PDE4DIP, or myomegalin, is a dual‐specificity AKAP known to colocalize with AKAP9 and PKA at the centrosome, which could play important roles in the localization and function of the AKAP/PKA complex in microtubule dynamics (Schmoker et al., 2018). The cAMP‐dependent PKA signaling pathway was reported to involve in many fundamental cellular processes in leukemia, including migration and proliferation (Murray & Insel, 2013; Xu et al., 2016). These studies hinted that *PDE4DIP* might play a role in leukemia by participating in the cAMP/PKA signaling pathway, but more convincing studies were needed to validate. Previously, *NOTCH2*, *FANCA*, *BCR*, and *ROS1* have been described to play an important role in leukemia. For example, Notch2 controlled nonautonomous Wnt‐signaling in CLL CLL (Mangolini, Gotte, & Moore, 2018). FANCA dysfunction might promote cytogenetic instability in adult acute myelogenous leukemia (Lensch et al., 2003). *BCR‐ABL1* fusion genes were leukemogenic, causing CML or ALL (Baccarani et al., 2019). *ROS1* revealed a central oncogenic role in CMML, which might represent a molecular target (Cilloni et al., 2013). In our study, we found *NOTCH2*, *FANCA*, *BCR*, and *ROS1* were significantly mutated in 109 Chinese patients with leukemia.

We used a methodology based on ACMG variant classification guidelines in 419 mutation genes, and identified 39 pathogenic and likely pathogenic mutations in 27 genes, which might be the cause of leukemia pathogenesis in these individuals. Particularly, there were 21 pathogenic or likely pathogenic gene in AML patients and 7 genes in CML patients, indicates that the pathogenesis of AML and CML might be more complicated. *PBRM1* (c.2819_2829del, p.L940fs) and *SUZ12* (c.1716_1717insG, p.L572fs) were only identified in ALL patients, *ALDH2* (rs540073928, p.A175D) and *FBXW7* (rs866987936, p.R361Q) only in CLL patients, and *CANT1* (c.407delT, p.L136fs) and *PAX8* (c.G201T, p.E67D) only in APL patients. These results suggested that the pathogenesis of different leukemia subgroups might be different. In addition, there were no studies of *CANT1*, *KIAA1549*, and *NFIB* on leukemia in previous literatures; therefore, the association between these genes and leukemia should be further investigated.

Subsequently, GO and KEGG analysis were performed to further understand the role of pathogenic mutation genes. GO annotation showed that the BP including gland development, leukocyte differentiation, respiratory system development, myeloid leukocyte differentiation, mesenchymal to epithelial transition, and so on were involved, which might provide further insight into the occurrence and development of leukemia. Moreover, the enriched KEGG pathway was found to be involved in leukemia (hsa05221 and hsa05220) and cancer‐related pathways (hsa05200, hsa05202, and hsa05230), which was consistent with findings from other previous studies on the pathogenesis of leukemia (McClure et al., 2018; de Noronha, Mitne‐Neto, & Chauffaille, 2017). Seven key genes (*CEBPA*, *FLT3*, *PAX5*, *PAX8*, *RUNX1*, *TP53*, and *WT1*) were obtained from PPIs network, most of which were reported to play a critical role in carcinogenesis and tumor progression (Junk et al., 2019; Rhodes, Vallikkannu, & Jayalakshmi, 2017; Slattery, Herrick, & Mullany, 2017).

Inevitably, this study has several drawbacks. First, this is a single‐center study, and a multi‐institutional large study will be necessary to verify the results. Second, due to insufficient data of leukemia patients, we could not evaluate the correlation of the mutations and clinical and prognostic data of leukemia. Third, the potential function and pathways were only predicted by bioinformatics and needed experimental verification.

## 5 CONCLUSION

Taken together, our study provided a map of gene mutations in Chinese patients with leukemia and enriched an understanding of the pathogenesis of leukemia. Of note, we are the first to demonstrate an association between *PDE4DIP* mutation and leukemia. Furthermore, we screened some pathogenic genes based on ACMG guidelines and performed GO analysis, pathway analysis, and PPI network. However, further investigations with larger cohorts and experimental research are warranted to further explore the potential mechanisms.

## CONFLICTS OF INTEREST

The authors declare that there are no conflicts of interest.

## AUTHOR CONTRIBUTIONS

The work presented here was carried out in collaboration between all authors. Hongxia Yao and Congming Wu carried out the molecular genetic studies and drafted the manuscript. Yueqing Chen, Li Guo, and Wenting Chen designed the methods and experiments, performed the statistical analyses and interpreted the results. Yanping Pan, Xiangjun Fu, and Guyun Wang collected clinical samples and information about patients. Hongxia Yao and Yipeng Ding conceived of the study, worked on associated data collection and their interpretation, participated in the design and coordination of the study, and funded the study. All authors read and approved the final manuscript.

## Supporting information

Fig S1

Fig S2

Table S1‐S4

## Data Availability

All the data regarding the findings are available within the manuscript. Anyone who is interested in the information should contact the corresponding author.
