# Supplementary figures and images for: Spectrum of gene mutations identified by targeted next‐generation sequencing in Chinese leukemia patients
Source: Mol Genet Genomic Med. 2020 Jul 7;8(9):e1369. doi: 10.1002/mgg3.1369 (PMC7507579; doi:10.1002/mgg3.1369)

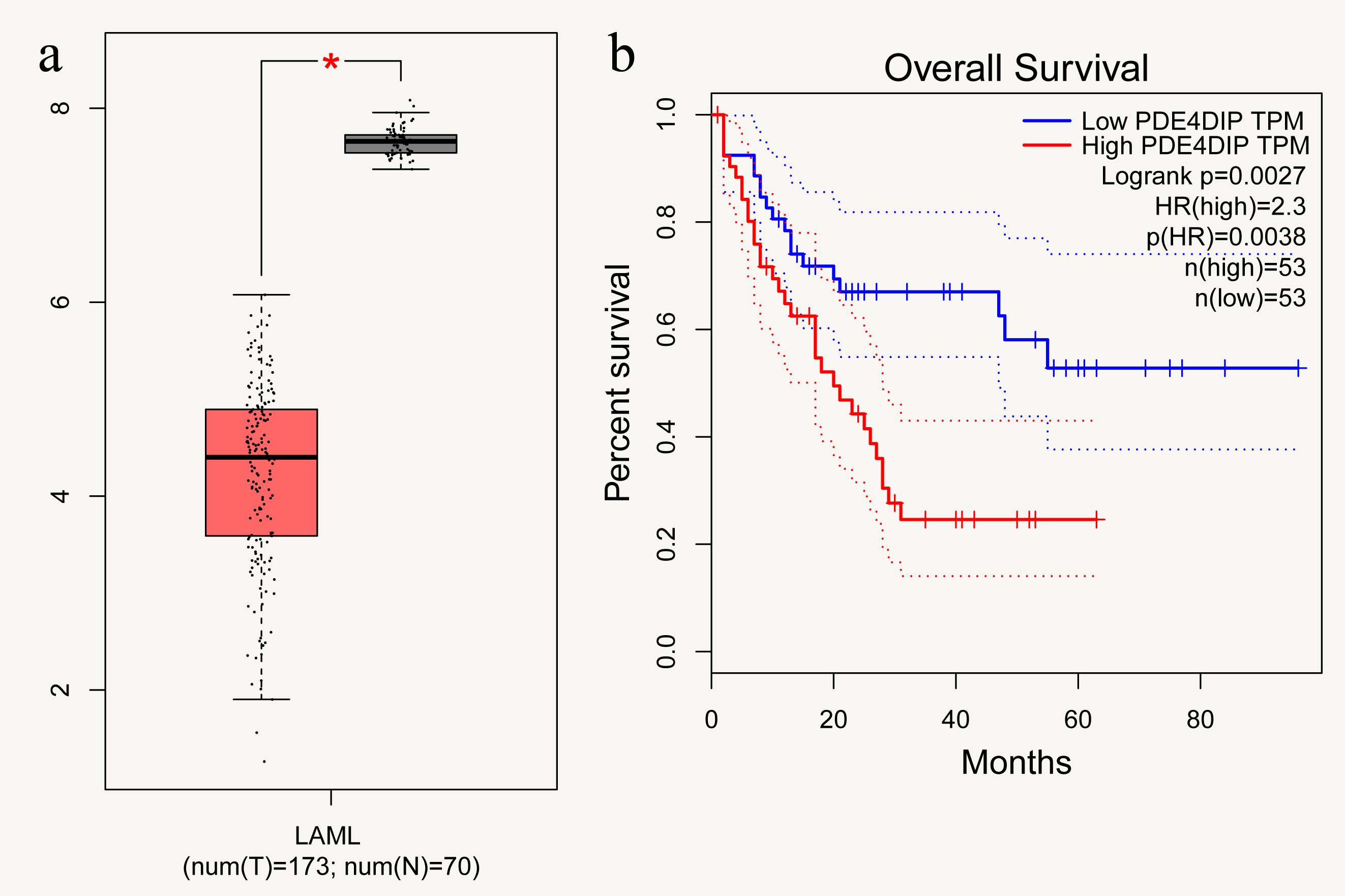

Supplement: Supplementary file 1 — Fig S1 [file MGG3-8-e1369-s001.tif]

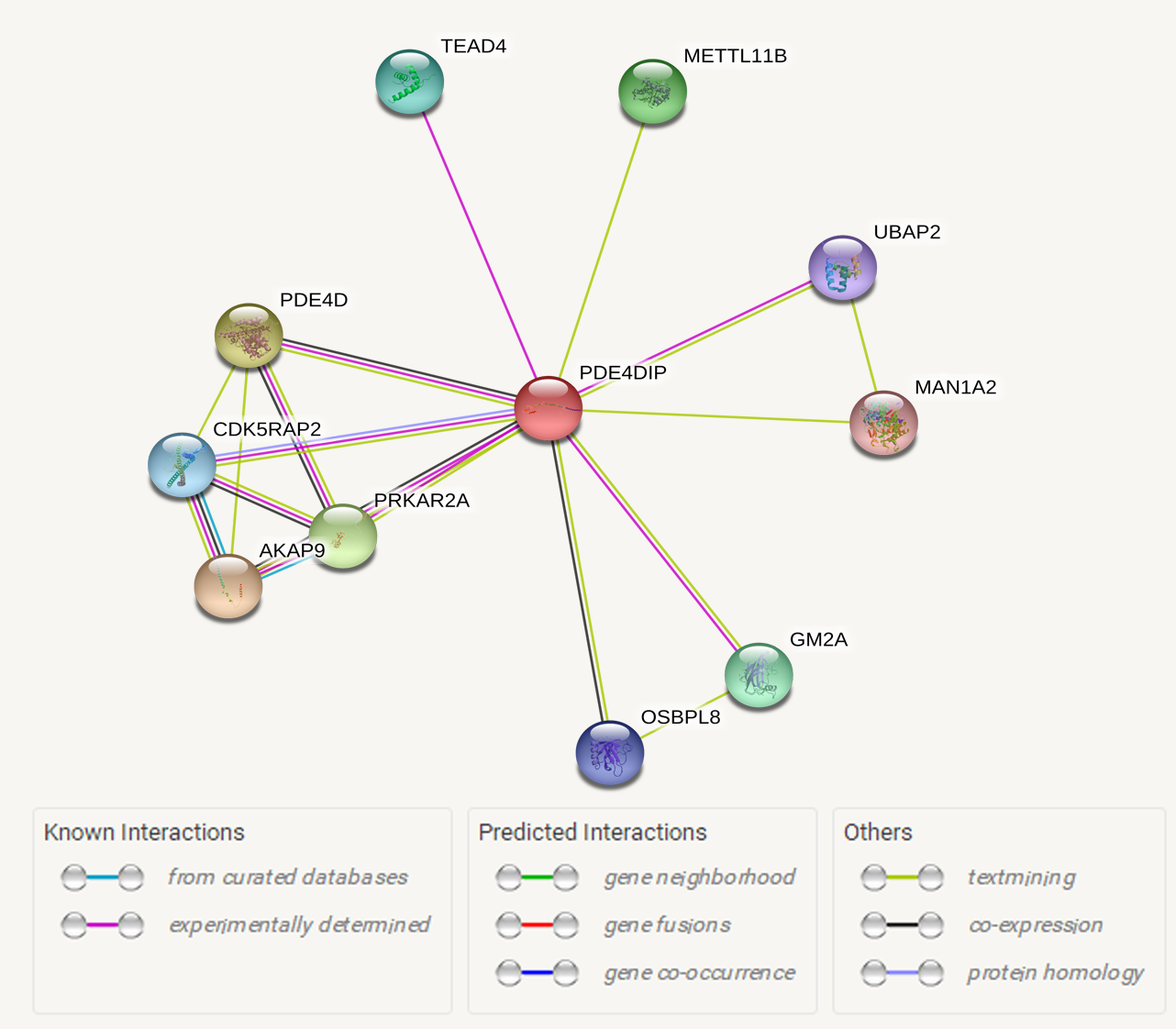

Supplement: Supplementary file 2 — Fig S2 [file MGG3-8-e1369-s002.tif]
