# Supplementary material for: Spectrum of gene mutations identified by targeted next‐generation sequencing in Chinese leukemia patients
Source: Mol Genet Genomic Med. 2020 Jul 7;8(9):e1369. doi: 10.1002/mgg3.1369 (PMC7507579; doi:10.1002/mgg3.1369)
Supplement: Supplementary file 3 — Table S1‐S4 [file MGG3-8-e1369-s003.docx]

**Supplementary Table 1. The 417 mutations genes in leukemia patients.**

| **Gene name** | | | | | | | | | |
| --- | --- | --- | --- | --- | --- | --- | --- | --- | --- |
| **Number of variants, (Cohort frequency)** | | | | | | | | | |
| PDE4DIP | FANCA | NOTCH2 | BCR | ROS1 | NACA | AKAP9 | NOTCH1 | COL1A1 | CAMTA1 |
| 120, (100.0%) | 52, (100.0%) | 51, (100.0%) | 50, (100.0%) | 49, (99.1%) | 47, (98.2%) | 42, (97.2%) | 40, (100.0%) | 39, (100.0%) | 38, (100.0%) |
| CLTCL1 | PCM1 | MYH11 | EP300 | BRCA2 | CIITA | ALK | USP6 | CARD11 | TSC2 |
| 38, (99.1%) | 37, (85.3%) | 37, (100.0%) | 36, (99.1%) | 35, (99.1%) | 35, (100.0%) | 33, (100.0%) | 33, (100.0%) | 32, (100.0%) | 32, (56.9%) |
| RECQL4 | KDM5A | NIN | TCF3 | KIAA1549 | CREBBP | SMARCA4 | MYH9 | FANCD2 | FGFR3 |
| 31, (100.0%) | 31, (89.0%) | 31, (100.0%) | 29, (95.4%) | 28, (100.0%) | 28, (65.1%) | 28, (80.7%) | 28, (100.0%) | 27, (89.9%) | 27, (99.1%) |
| TRIP11 | PDGFRB | MLLT4 | WRN | NUP214 | XPC | SETD2 | TET2 | NSD1 | ARHGEF12 |
| 27, (64.2%) | 26, (63.3%) | 26, (99.1%) | 26, (99.1%) | 26, (100.0%) | 25, (100.0%) | 25, (92.7%) | 25, (95.4%) | 25, (100.0%) | 25, (99.1%) |
| JAK3 | RET | NUMA1 | PCSK7 | FLT3 | NF1 | APC | ELN | CARS | ATM |
| 25, (91.7%) | 24, (99.1%) | 24, (98.2%) | 24, (100.0%) | 24, (100.0%) | 24, (100.0%) | 23, (99.1%) | 23, (98.2%) | 22, (98.2%) | 22, (69.7%) |
| ATIC | SRGAP3 | KDR | PMS2 | HIP1 | RALGDS | PER1 | PRDM16 | NTRK1 | PDGFRA |
| 21, (100.0%) | 21, (100.0%) | 21, (99.1%) | 21, (100.0%) | 21, (98.2%) | 21, (100.0%) | 21, (76.1%) | 20, (100.0%) | 20, (99.1%) | 20, (99.1%) |
| NUP98 | GOLGA5 | NTRK3 | CBFA2T3 | EML4 | CBLB | TSC1 | FGFR2 | SETBP1 | JAK1 |
| 20, (66.1%) | 20, (98.2%) | 20, (99.1%) | 20, (69.7%) | 19, (99.1%) | 19, (99.1%) | 19, (68.8%) | 19, (100.0%) | 19, (100.0%) | 18, (97.2%) |
| FCGR2B | MSH2 | EGFR | KTN1 | BUB1B | ASXL1 | PAX7 | DNMT3A | MET | CDH11 |
| 18, (67.0%) | 18, (94.5%) | 18, (100.0%) | 18, (88.1%) | 18, (65.1%) | 18, (100.0%) | 17, (90.8%) | 17, (88.1%) | 17, (97.2%) | 17, (100.0%) |
| ERCC2 | MKL1 | LPP | TFRC | WHSC1 | FIP1L1 | ECT2L | FGFR1OP | SMO | SYK |
| 17, (88.1%) | 17, (59.6%) | 16, (98.2%) | 16, (99.1%) | 16, (99.1%) | 16, (96.3%) | 16, (84.4%) | 16, (67.9%) | 16, (97.2%) | 16, (100.0%) |
| SUFU | LRIG3 | BLM | BRCA1 | ASPSCR1 | DNM2 | MUTYH | MSH6 | MLH1 | JAK2 |
| 16, (99.1%) | 16, (56.9%) | 16, (69.7%) | 16, (71.6%) | 16, (71.6%) | 16, (100.0%) | 15, (100.0%) | 15, (97.2%) | 15, (22.0%) | 15, (74.3%) |
| PICALM | SF3B1 | POU5F1 | NFKB2 | TCF7L2 | WT1 | MLLT6 | SH3GL1 | GNAS | TMPRSS2 |
| 15, (86.2%) | 14, (78.9%) | 14, (94.5%) | 14, (99.1%) | 14, (41.3%) | 14, (98.2%) | 14, (40.4%) | 14, (90.8%) | 14, (90.8%) | 14, (86.2%) |
| BCOR | NCOA1 | SLC34A2 | TERT | IL7R | PIK3R1 | RANBP17 | TFEB | TNFAIP3 | POT1 |
| 14, (100.0%) | 13, (99.1%) | 13, (88.1%) | 13, (87.2%) | 13, (100.0%) | 13, (95.4%) | 13, (93.6%) | 13, (95.4%) | 13, (97.2%) | 13, (94.5%) |
| ABL1 | NT5C2 | EXT2 | TSHR | AXIN1 | FUS | GAS7 | BRIP1 | BRD4 | ARID1A |
| 13, (97.2%) | 13, (99.1%) | 13, (98.2%) | 13, (100.0%) | 13, (56.9%) | 13, (99.1%) | 13, (95.4%) | 13, (99.1%) | 13, (22.9%) | 12, (19.3%) |
| NDRG1 | FANCG | FANCC | BRD3 | LMO1 | ARID2 | PML | ERBB2 | MSI2 | CIC |
| 12, (80.7%) | 12, (77.1%) | 12, (80.7%) | 12, (97.2%) | 12, (89.9%) | 12, (30.3%) | 12, (90.8%) | 12, (62.4%) | 12, (94.5%) | 12, (86.2%) |
| CBLC | ABL2 | TPR | PBRM1 | LIFR | IL6ST | ITK | ETV1 | FNBP1 | KIF5B |
| 12, (37.6%) | 11, (39.4%) | 11, (60.6%) | 11, (58.7%) | 11, (61.5%) | 11, (91.7%) | 11, (76.1%) | 11, (94.5%) | 11, (100.0%) | 11, (94.5%) |
| NCOA4 | AKT1 | ERCC4 | PALB2 | RNF43 | MAP2K2 | CNOT3 | ERG | TNFRSF14 | SFPQ |
| 11, (80.7%) | 11, (97.2%) | 11, (89.9%) | 11, (30.3%) | 11, (100.0%) | 11, (96.3%) | 11, (100.0%) | 11, (96.3%) | 10, (85.3%) | 10, (90.8%) |
| EPS15 | ARNT | RAF1 | BAP1 | FOXP1 | GMPS | IRF4 | RAC1 | IKZF1 | NCOA2 |
| 10, (99.1%) | 10, (82.6%) | 10, (20.2%) | 10, (17.4%) | 10, (99.1%) | 10, (71.6%) | 10, (72.5%) | 10, (100.0%) | 10, (81.7%) | 10, (90.8%) |
| BIRC3 | DDX10 | KCNJ5 | CDX2 | CDH1 | TP53 | ELL | EWSR1 | MED12 | RPL22 |
| 10, (68.8%) | 10, (85.3%) | 10, (100.0%) | 10, (95.4%) | 10, (93.6%) | 10, (100.0%) | 10, (40.4%) | 10, (98.2%) | 10, (29.4%) | 9, (89.9%) |
| ACSL3 | GATA2 | PIK3CA | EIF4A2 | BCL6 | KIT | NPM1 | EZR | JAZF1 | CREB3L2 |
| 9, (99.1%) | 9, (90.8%) | 9, (41.3%) | 9, (89.0%) | 9, (99.1%) | 9, (59.6%) | 9, (75.2%) | 9, (32.1%) | 9, (81.7%) | 9, (99.1%) |
| EXT1 | MLLT10 | MEN1 | MAML2 | TCF12 | LASP1 | STAT3 | GNA11 | AKT2 | SS18L1 |
| 9, (84.4%) | 9, (21.1%) | 9, (100.0%) | 9, (33.0%) | 9, (67.0%) | 9, (95.4%) | 9, (77.1%) | 9, (89.9%) | 9, (89.0%) | 9, (46.8%) |
| CRLF2 | KDM6A | ATRX | SEP6 | THRAP3 | BCL9 | MDM4 | PAX8 | FANCE | NFIB |
| 9, (71.6%) | 9, (71.6%) | 9, (62.4%) | 9, (#N/A) | 8, (99.1%) | 8, (90.8%) | 8, (91.7%) | 8, (19.3%) | 8, (85.3%) | 8, (15.6%) |
| CBL | ALDH2 | CRTC3 | TRAF7 | IL21R | RARA | CANT1 | STK11 | PPP2R1A | NF2 |
| 8, (97.2%) | 8, (51.4%) | 8, (33.9%) | 8, (15.6%) | 8, (25.7%) | 8, (14.7%) | 8, (86.2%) | 8, (97.2%) | 8, (22.0%) | 8, (80.7%) |
| ZRSR2 | BCL10 | RBM15 | PRCC | FBXO11 | MITF | EBF1 | DAXX | HMGA1 | HNRNPA2B1 |
| 8, (86.2%) | 7, (89.0%) | 7, (91.7%) | 7, (99.1%) | 7, (23.9%) | 7, (9.2%) | 7, (92.7%) | 7, (48.6%) | 7, (99.1%) | 7, (30.3%) |
| WHSC1L1 | PAX5 | SET | PRF1 | CCND1 | PAFAH1B2 | DDX6 | HOXC11 | WIF1 | RB1 |
| 7, (48.6%) | 7, (98.2%) | 7, (98.2%) | 7, (94.5%) | 7, (96.3%) | 7, (80.7%) | 7, (98.2%) | 7, (99.1%) | 7, (99.1%) | 7, (99.1%) |
| DICER1 | MAP2K1 | IDH2 | KLK2 | MPL | FUBP1 | FAM46C | PBX1 | C2orf44 | XPO1 |
| 7, (11.0%) | 7, (19.3%) | 7, (52.3%) | 7, (49.5%) | 6, (23.9%) | 6, (39.4%) | 6, (50.5%) | 6, (99.1%) | 6, (96.3%) | 6, (74.3%) |
| CHN1 | IDH1 | CTNNB1 | RPN1 | MLF1 | DEK | PRDM1 | BRAF | EZH2 | CD274 |
| 6, (80.7%) | 6, (4.6%) | 6, (97.2%) | 6, (92.7%) | 6, (57.8%) | 6, (48.6%) | 6, (50.5%) | 6, (43.1%) | 6, (90.8%) | 6, (4.6%) |
| CDKN2A | XPA | NR4A3 | KLF4 | CCDC6 | BMPR1A | FANCF | KRAS | HOXC13 | CDK4 |
| 6, (97.2%) | 6, (73.4%) | 6, (35.8%) | 6, (56.0%) | 6, (98.2%) | 6, (92.7%) | 6, (58.7%) | 6, (94.5%) | 6, (89.0%) | 6, (45.0%) |
| PTPN11 | BCL7A | LCP1 | CCNB1IP1 | GPHN | TNFRSF17 | CDK12 | CLTC | PRKAR1A | SRSF2 |
| 6, (5.5%) | 6, (78.9%) | 6, (99.1%) | 6, (53.2%) | 6, (65.1%) | 6, (99.1%) | 6, (5.5%) | 6, (85.3%) | 6, (96.3%) | 6, (100.0%) |
| MLLT1 | RUNX1 | ELF4 | SDHB | TRIM33 | TPM3 | NFE2L2 | PMS1 | PAX3 | FBXW7 |
| 6, (27.5%) | 6, (9.2%) | 6, (18.3%) | 5, (98.2%) | 5, (89.0%) | 5, (99.1%) | 5, (8.3%) | 5, (5.5%) | 5, (99.1%) | 5, (73.4%) |
| CD74 | PIM1 | MYB | CDK6 | VTI1A | POU2AF1 | FLI1 | ETV6 | ATF1 | SH2B3 |
| 5, (46.8%) | 5, (6.4%) | 5, (78.9%) | 5, (57.8%) | 5, (6.4%) | 5, (61.5%) | 5, (61.5%) | 5, (57.8%) | 5, (31.2%) | 5, (100.0%) |
| ETV4 | CD79B | ZNF521 | CCNE1 | TFPT | SMARCB1 | KDM5C | MDS2 | TAL1 | NRAS |
| 5, (42.2%) | 5, (78.0%) | 5, (60.6%) | 5, (43.1%) | 5, (79.8%) | 5, (33.9%) | 5, (80.7%) | 4, (65.1%) | 4, (70.6%) | 4, (3.7%) |
| MUC1 | ELK4 | BCL11A | TTL | ERCC3 | VHL | PPARG | FHIT | TFG | SBDS |
| 4, (97.2%) | 4, (62.4%) | 4, (99.1%) | 4, (73.4%) | 4, (7.3%) | 4, (6.4%) | 4, (56.0%) | 4, (82.6%) | 4, (99.1%) | 4, (24.8%) |
| PLAG1 | CHCHD7 | HEY1 | MYC | MLLT3 | OMD | HRAS | CREB3L1 | DDB2 | ZNF384 |
| 4, (18.3%) | 4, (37.6%) | 4, (75.2%) | 4, (18.3%) | 4, (47.7%) | 4, (11.9%) | 4, (54.1%) | 4, (17.4%) | 4, (99.1%) | 4, (8.3%) |
| MDM2 | TCL1A | CYLD | YWHAE | TAF15 | DDX5 | CD79A | BCL3 | ZNF331 | U2AF1 |
| 4, (56.0%) | 4, (54.1%) | 4, (92.7%) | 4, (20.2%) | 4, (21.1%) | 4, (4.6%) | 4, (3.7%) | 4, (15.6%) | 4, (88.1%) | 4, (21.1%) |
| CHEK2 | WAS | TFE3 | SLC45A3 | FH | MYCN | REL | HOXD13 | CREB1 | FEV |
| 4, (23.9%) | 4, (8.3%) | 4, (28.4%) | 3, (2.8%) | 3, (7.3%) | 3, (2.8%) | 3, (74.3%) | 3, (19.3%) | 3, (3.7%) | 3, (35.8%) |
| ETV5 | RAP1GDS1 | TRIM27 | CCND3 | GOPC | HOXA9 | HOXA11 | FGFR1 | LMO2 | DDIT3 |
| 3, (2.8%) | 3, (50.5%) | 3, (89.9%) | 3, (80.7%) | 3, (13.8%) | 3, (30.3%) | 3, (1.8%) | 3, (8.3%) | 3, (99.1%) | 3, (48.6%) |
| HMGA2 | MAX | MAF | MAP2K4 | SUZ12 | BCL2 | TPM4 | SDC4 | PDGFB | P2RY8 |
| 3, (90.8%) | 3, (2.8%) | 3, (4.6%) | 3, (4.6%) | 3, (3.7%) | 3, (43.1%) | 3, (97.2%) | 3, (97.2%) | 3, (86.2%) | 3, (2.8%) |
| SSX1 | GATA1 | GPC3 | LCK | RPL5 | SDHC | MYD88 | FOXL2 | IL2 | TLX3 |
| 3, (67.0%) | 3, (5.5%) | 3, (2.8%) | 2, (1.8%) | 2, (1.8%) | 2, (54.1%) | 2, (1.8%) | 2, (1.8%) | 2, (63.3%) | 2, (4.6%) |
| HIST1H3B | HIST1H4I | TCEA1 | COX6C | GNAQ | LHFP | BCL11B | CBFB | SMARCE1 | MALT1 |
| 2, (94.5%) | 2, (2.8%) | 2, (1.8%) | 2, (29.4%) | 2, (42.2%) | 2, (2.8%) | 2, (43.1%) | 2, (6.4%) | 2, (42.2%) | 2, (23.9%) |
| FSTL3 | LYL1 | OLIG2 | MSN | RPL10 | CDKN2C | JUN | H3F3A | HOXD11 | SOX2 |
| 2, (1.8%) | 2, (1.8%) | 2, (31.2%) | 2, (3.7%) | 2, (96.3%) | 1, (11.0%) | 1, (0.9%) | 1, (1.8%) | 1, (0.9%) | 1, (0.9%) |
| PHOX2B | CHIC2 | HOOK3 | TAL2 | GATA3 | PTEN | SDHD | CCND2 | BTG1 | SOCS1 |
| 1, (21.1%) | 1, (0.9%) | 1, (0.9%) | 1, (0.9%) | 1, (98.2%) | 1, (14.7%) | 1, (0.9%) | 1, (8.3%) | 1, (0.9%) | 1, (0.9%) |
| HERPUD1 | HLF | SS18 | MAFB | TOP1 | MN1 | PHF6 |  |  |  |
| 1, (22.0%) | 1, (0.9%) | 1, (0.9%) | 1, (0.9%) | 1, (0.9%) | 1, (2.8%) | 1, (17.4%) |  |  |  |

**Supplementary** **Table 2. GO-term for PDE4DIP based on STRING database**

| **GO-term** | **Description** | **Count in gene set** | **False discovery rate** | **Gene name** |
| --- | --- | --- | --- | --- |
| **Biological Process (GO)** | | | | |
| GO:0031116 | positive regulation of microtubule polymerization | 3 of 27 | 0.00036 | AKAP9,CDK5RAP2,PDE4DIP |
| GO:1903358 | regulation of Golgi organization | 2 of 15 | 0.0044 | AKAP9,PDE4DIP |
| GO:0086004 | regulation of cardiac muscle cell contraction | 2 of 36 | 0.0121 | AKAP9,PDE4D |
| GO:0071320 | cellular response to cAMP | 2 of 54 | 0.0197 | AKAP9,PDE4D |
| GO:0000226 | microtubule cytoskeleton organization | 3 of 393 | 0.0429 | AKAP9,CDK5RAP2,PDE4DIP |
| GO:0002027 | regulation of heart rate | 2 of 95 | 0.044 | AKAP9,PDE4D |
| GO:0097711 | ciliary basal body-plasma membrane docking | 2 of 95 | 0.044 | AKAP9,CDK5RAP2 |
| **Molecular Function (GO)** | | | | |
| GO:0030552 | cAMP binding | 2 of 22 | 0.0087 | PDE4D,PRKAR2A |
| GO:0051018 | protein kinase A binding | 2 of 50 | 0.0139 | AKAP9,PRKAR2A |
| GO:0032947 | protein-containing complex scaffold activity | 2 of 68 | 0.0188 | AKAP9,PDE4DIP |
| GO:0004553 | hydrolase activity, hydrolyzing O-glycosyl compounds | 2 of 99 | 0.0312 | GM2A,MAN1A2 |
| GO:0005319 | lipid transporter activity | 2 of 134 | 0.0378 | GM2A,OSBPL8 |
| GO:0044325 | ion channel binding | 2 of 120 | 0.0378 | AKAP9,PDE4D |
| **Cellular Component (GO)** | | | | |
| GO:0005813 | centrosome | 5 of 468 | 0.00048 | AKAP9,CDK5RAP2,PDE4D,PDE4DIP,PRKAR2A |

The data were extracted from the STRING online software (https://string-db.org/).

**Supplementary** **Table 3. The top 50 significantly different genes in leukemia subgroups.**

| **Genes** | **Frequencies** | | | | | **Genes** | **Frequencies** | | | | |
| --- | --- | --- | --- | --- | --- | --- | --- | --- | --- | --- | --- |
|  | **ALL** | **APL** | **CML** | **AML** | **CLL** |  | **ALL** | **APL** | **CML** | **AML** | **CLL** |
| DDIT3 | 68.4% | 41.7% | 35.7% | 63.3% | 12.5% | HNRNPA2B1 | 42.1% | 33.3% | 39.3% | 10.0% | 50.0% |
| TCF12 | 57.9% | 75.0% | 89.3% | 40.0% | 87.5% | FANCF | 68.4% | 58.3% | 50.0% | 53.3% | 87.5% |
| WHSC1L1 | 73.7% | 62.5% | 35.7% | 40.0% | 25.0% | HLF | 78.9% | 100.0% | 60.7% | 73.3% | 87.5% |
| HEY1 | 84.2% | 66.7% | 75.0% | 86.7% | 37.5% | TTL | 68.4% | 83.3% | 85.7% | 63.3% | 50.0% |
| CHCHD7 | 31.6% | 25.0% | 39.3% | 40.0% | 75.0% | ATF1 | 63.2% | 50.0% | 53.6% | 56.7% | 25.0% |
| CDK4 | 73.7% | 37.5% | 46.4% | 26.7% | 62.5% | FAM46C | 57.9% | 41.7% | 67.9% | 33.3% | 62.5% |
| HOXD13 | 15.8% | 50.0% | 0.0% | 16.7% | 12.5% | REL | 63.2% | 79.2% | 78.6% | 66.7% | 100.0% |
| PHF6 | 5.3% | 12.5% | 28.6% | 10.0% | 50.0% | NCOA4 | 89.5% | 62.5% | 89.3% | 76.7% | 100.0% |
| ATM | 63.2% | 95.8% | 64.3% | 66.7% | 100.0% | XPO1 | 73.7% | 87.5% | 82.1% | 53.3% | 87.5% |
| PIK3CA | 36.8% | 50.0% | 42.9% | 26.7% | 75.0% | ALDH2 | 42.1% | 58.3% | 50.0% | 60.0% | 25.0% |
| SDHC | 84.2% | 70.8% | 50.0% | 50.0% | 87.5% | FEV | 26.3% | 50.0% | 39.3% | 33.3% | 62.5% |
| NUP98 | 73.7% | 66.7% | 89.3% | 46.7% | 50.0% | XPA | 94.7% | 70.8% | 78.6% | 60.0% | 62.5% |
| MDS2 | 84.2% | 58.3% | 64.3% | 70.0% | 37.5% | GNAQ | 31.6% | 66.7% | 35.7% | 40.0% | 37.5% |
| SS18L1 | 52.6% | 79.2% | 50.0% | 56.7% | 87.5% | JAZF1 | 100.0% | 83.3% | 75.0% | 70.0% | 100.0% |
| SMARCE1 | 26.3% | 41.7% | 60.7% | 30.0% | 62.5% | TAF15 | 73.7% | 54.2% | 42.9% | 53.3% | 37.5% |
| ETV6 | 57.9% | 62.5% | 39.3% | 76.7% | 37.5% | BUB1B | 68.4% | 66.7% | 64.3% | 70.0% | 37.5% |
| BCL11B | 63.2% | 54.2% | 28.6% | 40.0% | 25.0% | TFPT | 100.0% | 79.2% | 82.1% | 63.3% | 87.5% |
| NR4A3 | 31.6% | 50.0% | 21.4% | 46.7% | 12.5% | TRIP11 | 84.2% | 66.7% | 60.7% | 50.0% | 75.0% |
| IL21R | 42.1% | 33.3% | 17.9% | 23.3% | 0.0% | COX6C | 15.8% | 50.0% | 21.4% | 30.0% | 25.0% |
| HRAS | 52.6% | 58.3% | 50.0% | 50.0% | 87.5% | KLK2 | 31.6% | 45.8% | 64.3% | 53.3% | 37.5% |
| ELF4 | 10.5% | 41.7% | 10.7% | 16.7% | 0.0% | CD79B | 68.4% | 87.5% | 75.0% | 73.3% | 100.0% |
| GPHN | 78.9% | 58.3% | 64.3% | 70.0% | 37.5% | YWHAE | 31.6% | 20.8% | 10.7% | 26.7% | 0.0% |
| PER1 | 78.9% | 79.2% | 64.3% | 90.0% | 50.0% | CBFA2T3 | 89.5% | 58.3% | 75.0% | 63.3% | 62.5% |
| MLLT10 | 10.5% | 37.5% | 10.7% | 30.0% | 0.0% | TSC1 | 84.2% | 50.0% | 71.4% | 73.3% | 75.0% |
| IL2 | 84.2% | 70.8% | 46.4% | 63.3% | 50.0% | SSX1 | 52.6% | 66.7% | 57.1% | 83.3% | 75.0% |

AML, acute myeloid leukemia; ALL, acute lymphoblastic leukemia; APL, acute promyelocytic leukemia; CML, chronic myeloid leukemia; CLL, chronic lymphocytic leukemia.

**Supplementary** **Table 4. The pathologic mutations for** **leukemia patients**

| **Number** | **Leukemia type** | **Pathogenic mutations unmber** | **Pathogenic mutations** |
| --- | --- | --- | --- |
| D021 | ALL | 1 | PBRM1, 3:52623221, .GTAAGCCTGAGA/G |
| D054 | ALL | 1 | SMO, 7:128848655, .,G/C |
| D113 | ALL | 2 | FBXW7, 4:153249385, rs867384286,G/A  NRAS, 1:115258747, rs121913237,C/T |
| D177 | ALL | 2 | SUZ12, 17:30322703, .,A/AG  TFRC, 3:195791279, rs184956956,C/T |
| D002 | AML | 1 | ATIC, 2:216182882, rs575560797A/T |
| D022 | AML | 1 | NRAS, 1:115258744, rs121434596C/A |
| D023 | AML | 1 | IDH1, 2:209113113, rs121913499G/C |
| D028 | AML | 2 | FLT3, 13:28592642, rs121913488,C/A  SETD2, 3:47155452, .,C/CCGGTCCAA |
| D029 | AML | 2 | MYH9, 22:36715582, rs372016779,T/C  TP53, 17:7578406, rs28934578,C/T |
| D031 | AML | 1 | FLT3, 13:28592640, rs121913487,A/C |
| D040 | AML | 3 | CEBPA, 19:33793130, .,AT/A  WT1, 11:32417909, .,C/CGACA  WT1, 11:32417942, .,A/AG |
| D050 | AML | 2 | NRAS, 1:115256529, rs11554290,T/G  SMO, 7:128846423, .,T/C |
| D075 | AML | 1 | CANT1, 17:76993297, .,CA/C |
| D078 | AML | 3 | ASXL1, 20:31021634, .,C/CA  CBL, 11:119155730, .,C/CCGCGCTTTCTT  PAX5, 9:36966685, .,AGCGAGTG/A |
| D080 | AML | 1 | ALDH2, 12:112228350, rs540073928,C/A |
| D082 | AML | 1 | KRAS, 12:25398281, rs112445441,C/T |
| D163 | AML | 2 | ALDH2, 12:112228350, rs540073928,C/A  CEBPA, 19:33793153, .,GCAGATGCCGCC/G |
| D183 | AML | 1 | KIAA1549, 7:138602332, .,TGA/T |
| D47 | AML | 1 | ALDH2, 12:112228350, rs540073928,C/A |
| D53 | AML | 1 | CEBPA, 19:33793092, .,A/ACT |
| D037 | APL | 2 | WT1, 11:32413557, .,G/T  WT1, 11:32417920, .,G/GAGTCGGGGCTACTCCAGGC |
| D051 | APL | 1 | FLT3, 13:28592642, rs121913488,C/A |
| D066 | APL | 1 | TFRC, 3:195791279, rs184956956,C/T |
| D072 | APL | 1 | TFRC, 3:195791279, rs184956956,C/T |
| D081 | APL | 1 | PAX8, 2:114002192, .,C/A |
| D058 | CLL | 1 | ALDH2, 12:112228350, rs540073928,C/A |
| D005 | CLL | 1 | FBXW7, 4:153247366, rs866987936C/T |
| D056 | CML | 2 | FOXP1, 3:71037162, .,TG/T  RUNX1, 21:36171607, .,G/A |
| D059 | CML | 2 | CBL, 11:119148892, rs387906666,A/G  SETBP1, 18:42531913, rs267607040,G/A |
| D173 | CML | 1 | TFRC, 3:195785460, rs772017482,T/C |
| D180 | CML | 1 | FANCG, 9:35077398, rs376732298,T/TG |
| D004 | CML | 2 | NFIB, 9:14398522, .,C/A  RUNX1, 21:36171607, G/A |
